# *Ganoderma* triterpenoids attenuate tumour angiogenesis in lung cancer tumour-bearing nude mice

**DOI:** 10.1080/13880209.2020.1839111

**Published:** 2020-11-08

**Authors:** Wei Liu, Ruiying Yuan, Aihua Hou, Song Tan, Xin Liu, Pengcheng Tan, Xiaoming Huang, Jinguo Wang

**Affiliations:** a Department of Oncology, Yantai Hospital of Traditional Chinese Medicine, Yantai, Shandong, China; b Medical College, Tibet University, Lasa, China

**Keywords:** Antitumor activity, gefitinib, apoptosis

## Abstract

**Context:**

*Ganoderma lucidum* (Leyss. ex Fr.) Karst. (Polyporaceae) triterpenoids (GLTs), the main components and bioactive metabolites of *G. lucidum*, have antitumour activity.

**Objective:**

We investigated the effects of GLTs in lung cancer tumour-bearing nude mice and their potential mechanism.

**Materials and methods:**

Forty BALB/c nude mice were randomly divided into four groups: saline control, GLT (1 g/kg/day), gefitinib (GEF, 15 mg/kg/day), and GLT (1 g/kg/day) + GEF (15 mg/kg/day) for 14 days. Cell viability was conducted using the Cell Counting Kit-8 assay. The tumour volume, inhibition rate, histopathological, microvessel density (MVD), mRNAs, and proteins were determined.

**Results:**

GLTs inhibited the cell viability of A549 cells with an IC_50_ value of 14.38 ± 0.29 mg/L, while the IC_50_ value of GEF was 10.26 ± 0.47 μmol/L. The tumour inhibition rate in the GLT + GEF group (51.54%) was significantly decreased relative to the saline control… group (*p* < 0.05). The MVD in the GLT + GEF group (2.9 ± 0.7) was significantly decreased than that in the saline control group (12.8 ± 1.4, *p* < 0.05). The angiostatin, endostatin, and Bax protein expression in the GLT, GEF, and GLT + GEF groups were significantly increased compared to those in the saline control group, while the VEGFR2 and Bcl-2 protein expression were decreased.

**Discussion and conclusions:**

Our study provided evidence that GLT and GEF combination therapy may be a promising candidate for the treatment of lung cancer and as an experimental basis for clinical treatment.

## Introduction

Lung cancer involves malignant lung tumours that originate in the bronchial epithelium cells. It is characterized by uncontrolled cell growth in lung tissue, which may cause metastasis, invasion of adjacent tissues, and infiltration outside the lungs. Lung cancer is divided into small cell lung cancer (SCLC) and non-small cell lung cancer (NSCLC) (Lecharpentier et al. 2011; Aberle et al. 2011; Wen et al. 2017). It is the most frequent cancer among all malignant tumours worldwide, causing cancer-related deaths of 1.6 million individuals every year (Liu et al. 2017). In addition, lung cancer is the primary cause of cancer-related deaths among men and second-leading cause of cancer-related deaths among women worldwide. In recent years, research on treatments of lung cancer have led to many advances in surgery, chemotherapy, and radiotherapy (Wang et al. 2018). Chemotherapy and radiotherapy are effective therapeutic methods for cancer in the clinic. However, there are adverse reactions and side effects of chemotherapy and radiotherapy. Many biologically active metabolites derived from plants, fungi, algae, and animals have been demonstrated to be effective in terms of anticancer effects and have minimal adverse reactions and side effects (Yin et al. 2013; Natan and Banin 2017).

*Ganoderma lucidum* (Leyss. ex Fr.) Karst. (Polyporaceae), a traditional Chinese medicine, may treat and prevent many diseases. The medicinal active ingredients isolated from *G. lucidum* include alkaloids, ganoderic acids, methyl genoderates, ganorderenic acids, derivatized hydroquinones, and *G. lucidum* triterpenoids, which have demonstrated a variety of bioactivities, such as antitumor, antioxidant, anti-acetylcholinesterase, and anti-inflammatory activities (Chen et al. 2016; Hu et al. 2016). Some studies have suggested that *G. lucidum* triterpenoids (GLTs) are ideal candidates for anticancer treatment (Kohno et al. 2017). Gefitinib (GEF) is mainly used for the treatment of patients with advanced NSCLC. Nevertheless, most patients in whom treatment is effective develop acquired drug resistance after taking gefitinib for 10 to 16 months. Therefore, identifying a way to overcome drug resistance has become one of the urgent problems to be solved in the field of molecular-targeted therapy (Costa et al. 2008).

In the current study, we established the tumour-bearing nude mouse model to assess whether GLTs increase the tumour suppressive effect of GEF in A549 cells *in vivo* and clarify its main potential mechanism to provide new options for the treatment of lung cancer in clinical therapy.

## Materials and methods

### Animals

Forty BALB/c nude mice (female, 4–6 weeks of age) were purchased from Changzhou Cavens Laboratory Animal Co., Ltd. (animal production certificate number: SCXK (Su) 20160010). The mice were housed (5 mice/cage) in individually ventilated cages and renewed every 24 h under a 12 h light/dark cycle at around 20–26 °C with daily temperature differences ≤4 °C and a relative humidity of 40–70% with free access to food and water during the quarantine and experimental periods. All experiments were conducted in accordance with the Guide for the Care and Use of Laboratory Animals of the National Institutes of Health (National Research Council (US) Institute for Laboratory Animal Research 1996) and were approved by Yantai Hospital of Traditional Chinese Medicine Animal Ethics Committee.

### Cell counting kit-8 assay

A549 (human lung cancer cells) and BEAS-2B (human normal lung epithelial cells) cell lines (ATCC, USA) were cultured. Cell viability was measured using the Cell Counting Kit-8 (CCK-8) assay. Briefly, 5 × 10^3^ cells were cultured in 96-well plates. After 24 h, the cells were treated with the indicated concentrations of GEF (51, 01, 52, 02, 530 μmol/L) and GLT (0, 2.5, 5, 10, 20, 40, 80 mg/L). Cells were evaluated finally using the CCK-8 assay (MedChem Express, USA). The absorbance was measured at 450 nm. The growth inhibition rate and IC_50_ were calculated.

### Tumour-bearing nude mouse model

A549 cells were cultured using the adherent method, and the cells were grown in the logarithmic phase and mixed with serum-free Dulbecco's Modified Eagle Medium (Gibco, Grand Island, NY, USA) to a final suspension of 5 × 10^7^/mL. The cell suspensions (0.1 mL) were injected subcutaneously on the right side in nude mice, and the tumour-bearing mouse model was established after 14 days with tumour formation.

### Animal grouping and administration

The tumour-bearing mice were randomly divided into four groups (10 in each group): saline control group, GLT group (Sigma, 1 g/kg), gefitinib group (GEF, AstraZeneca Company, UK, 15 mg/kg), and GLT (1 g/kg) + GEF group (15 mg/kg). Mice in the saline control group were injected and received intragastric administration with the same amount of saline. The GLT group received intragastric administration and GEF group underwent intraperitoneal injections. When the tumour diameter reached 6-8 mm, administration was initiated once a day for 14 consecutive days. On days 1, 8, and 15, the body weights of the mice were recorded. Furthermore, we included baseline control mice (n = 10), which were only used as a weight reference, and no other experiments were conducted with these mice.

### Tumour volume measurement

The tumour length (long diameter, a) and width (short diameter, b) of tumour growth were observed every third day one time before and after treatment. The tumour volume (V) was calculated using the following formula: V = (a × b^2^)/2. The mice were sacrificed under aseptic conditions, and simultaneously, the tumour metastases were finely dissected, separated, processed, and frozen in liquid nitrogen.

### Tumour growth inhibition rate

Twenty-four hours after the last treatment, the mice were sacrificed and the tumour bodies were isolated. The tumour was weighed, and the growth inhibition rate of the tumour was calculated using the following formula: Growth inhibition rate of tumour = (average tumour volume in model group - average tumour volume in treated group)/average tumour volume in model group × 100%.

### Haematoxylin and eosin staining

Briefly, tumour tissues were fixed in 4% paraformaldehyde for 24 h, washed with phosphate-buffered saline three times, perfused with haematoxylin for 15 min, differentiated by 1% hydrochloride-ethanol, and perfused in eosin for another 15 min [haematoxylin and eosin (H&E), Beijing Solarbio Science & Technology Co., Ltd., China]. The pathological changes in the tumour tissues were observed under optical microscopy (magnification, × 100; Olympus Corporation).

### Immunohistochemical assessments

The tumour tissues were embedded in paraffin, sectioned, and were subjected to xylene dewaxing. Citric acid was used for antigen retrieval. A 3% H_2_O_2_-methanol solution was used to block endogenous peroxidase for 15 min, followed by blocking with 5% bovine serum albumin for 20 min. The primary anti-cluster of differentiation (CD)31 polyclonal antibody (1:50, ab28364, Abcam, UK) was added dropwise at 37 °C and incubated for 2 h. Goat anti-rabbit immunoglobulin G (IgG; Proteintech, USA) labelled with horseradish peroxidase was incubated at 37 °C for 30 min. 3, 3-diaminobenzidine (Solarbio, Beijing, China) was used to develop colour, and haematoxylin was used for counterstaining; the sections were then dehydrated, rendered transparent, and cover-slipped with Permount mounting medium (Thermo Fisher Scientific, Inc., USA). The sections were observed under a × 100 optical microscope (Olympus, Japan) to detect the positive protein expression in the tumour tissue.

Microvascular density (MVD) quantitative analysis was performed according to a method proposed previously (Weidner et al. 1992). In each section, the number of microvessels stained with CD31 was counted in five fields, and the average value was used as the MVD value of the tumour tissue.

### Quantitative reverse-transcription polymerase chain reaction

A TRIzol kit (Invitrogen, Carlsbad, CA, USA) was used to extract total RNA from tumour tissues. RNAs were reverse-transcribed to cDNA using SuperScript III Reverse Transcriptase (Thermo Fisher Scientific, Waltham, MA, USA). Polymerase chain reaction assays were performed using the Mastercycler ep realplex2 system (Eppendorf, Hamburg, Germany) with the following conditions: 95 °C for 30 s, 95 °C for 5 s, and 60 °C for 45 s (40 cycles). The primer sequences are listed in Table 1. Data analysis was carried out using the 2^−ΔΔCt^ method, and glyceraldehyde 3-phosphate dehydrogenase (GAPDH) mRNA was used as the internal control.

**Table 1.** Primer sequences (Shanghai Shenggong Biological Engineering Technology Service Co., Ltd.).

Gene	Primer sequence
Angiostatin	forward 5′ -CCCAACATGGACCATAAGGAAGT-3′
	reverse 5′-TGTGGGCAATTCCACAACACTC-3′
Endostatin	forward 5′-CCGGAATTCATGCACAGCCACCGCGACTTCCAG-3′
	reverse 5′-GCCGGATCCCTACTTGGAGGCAGTCATGAAGCTGTT-3′
GAPDH	forward 5′-ATGTTCGTCATG GGTGTGAA-3′
	reverse 5′-TGTGGTCATGAGTCCTTCCA-3′

### Western blot assay

The total protein of the tumour tissues was extracted using radioimmunoprecipitation assay lysis buffer (Beyotime, Shanghai, China) containing proteinase inhibitor cocktail, and the concentration of protein was determined using a bicinchoninic acid assay. Proteins (50 µg) were separated by 10% sodium dodecyl sulphate-polyacrylamide gel electrophoresis and then transferred onto polyvinylidene fluoride membranes (EMD Millipore, USA). The membranes were blocked with 5% skimmed milk at 4 °C overnight and incubated with primary antibodies against VEGFR2 (1:1,000; ab11939, Abcam), angiostatin (1:2,000; ab2904, Abcam), endostatin (1:500; ab202973, Abcam), Bcl-2 (1:1,000; ab32124, Abcam), and Bax (1:2,000; ab32503, Abcam) at 4 °C overnight. After incubating with sheep anti-rabbit IgG (1:5,000; ab97095; Abcam) at 37 °C for 1 h, protein bands were visualised using the enhanced chemiluminescence system (Thermo Fisher Scientific, Inc.). Protein expression levels were normalised to GAPDH expression (1:10,000; ab181602; Abcam) and quantified using ImageJ software version 1.46 (NIH).

### Statistical analysis

SPSS Statistics v20.0 software (IBM Corp.) was used for statistical analysis. All the data are expressed as means ± standard deviation. Comparisons between the two groups were performed using Student’s *t*-test, and multiple groups were compared using one-way analysis of variance followed by Fisher’s LSD *post hoc* test. *p* < 0.05 was considered to indicate a statistically significant difference.

## Results

### GLTs inhibited the proliferation of NSCLC cells

**Figure 1.**
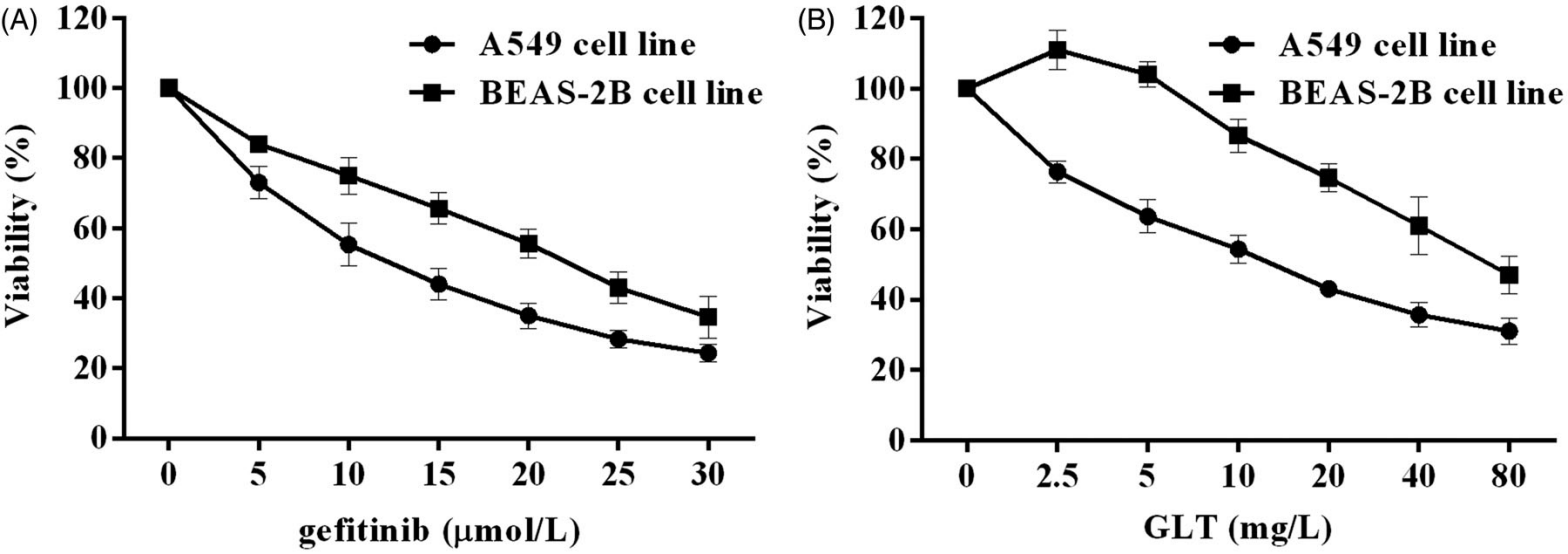
GEF and GLT inhibited the cell growth in A549 and BEAS-2B cells. A Cell viability (%) of A549 and BEAS-2B cells treated with gefitinib for 48 h; B. Cell viability (%) of A549 and BEAS-2B cells treated with GLT for 48 h.

To examine the cytotoxic effects of GLT and GEF, a CCK-8 assay was conducted. As shown in Figure 1, GEF and GLT inhibited the proliferation of the A549 cell lines in a concentration-dependent manner. The IC_50_ values of GEF and GLT at 48 h of treatment for the A549 cell lines were 10.26 ± 0.47 μmol/L and 14.38 ± 0.29 mg/L, respectively. However, GLTs showed a much less potent cytotoxic effect on the BEAS-2B cell line, with an IC_50_ value of 78.62 ± 2.53 mg/L.

### General status and weight of the mice

On days 8 and 15 after drug administration, the average weights of the mice in the GEF group were 18.80 ± 0.61 and 18.25 ± 1.19 g, respectively; these were significantly lower than those in the baseline control group (*p* < 0.05) (Table 2). Simultaneously, the weight difference between the GLT + GEF and GLT groups was not statistically significant on day 8, while on day 15, the weight of the mice in the GLT + GEF group was lower than that in the saline control group (*p* < 0.05). The mice in the GEF group showed signs of agitation and drowsiness, reduced physical activity, decreased feeding, and dull skin colour, which may have been the side effects of GEF. The mice in the GLT + GEF group showed no obvious agitation or drowsiness, accessed water and food without difficulty, and exhibited an ameliorated skin colour compared to those of mice in the GEF group. The results indicated that GLTs alleviated the side effects caused by GEF.

**Table 2.** The average weight of the mice in each group (g).

Group	1 day	8 days	15 days
Baseline control	16.43 ± 0.49	20.12 ± 0.58	22.45 ± 1.15
Saline control	16.61 ± 0.62	18.65 ± 0.96*	20.09 ± 1.87*
GLT	16.81 ± 0.69	18.95 ± 0.72*	19.23 ± 1.26*
GEF	16.75 ± 0.98	18.80 ± 0.61*	18.25 ± 1.19*
GLT + GEF	15.91 ± 0.92	19.88 ± 1.45	21.18 ± 1.47

vs baseline control group *p < 0.05.

### Tumour volume changes

**Figure 2.**
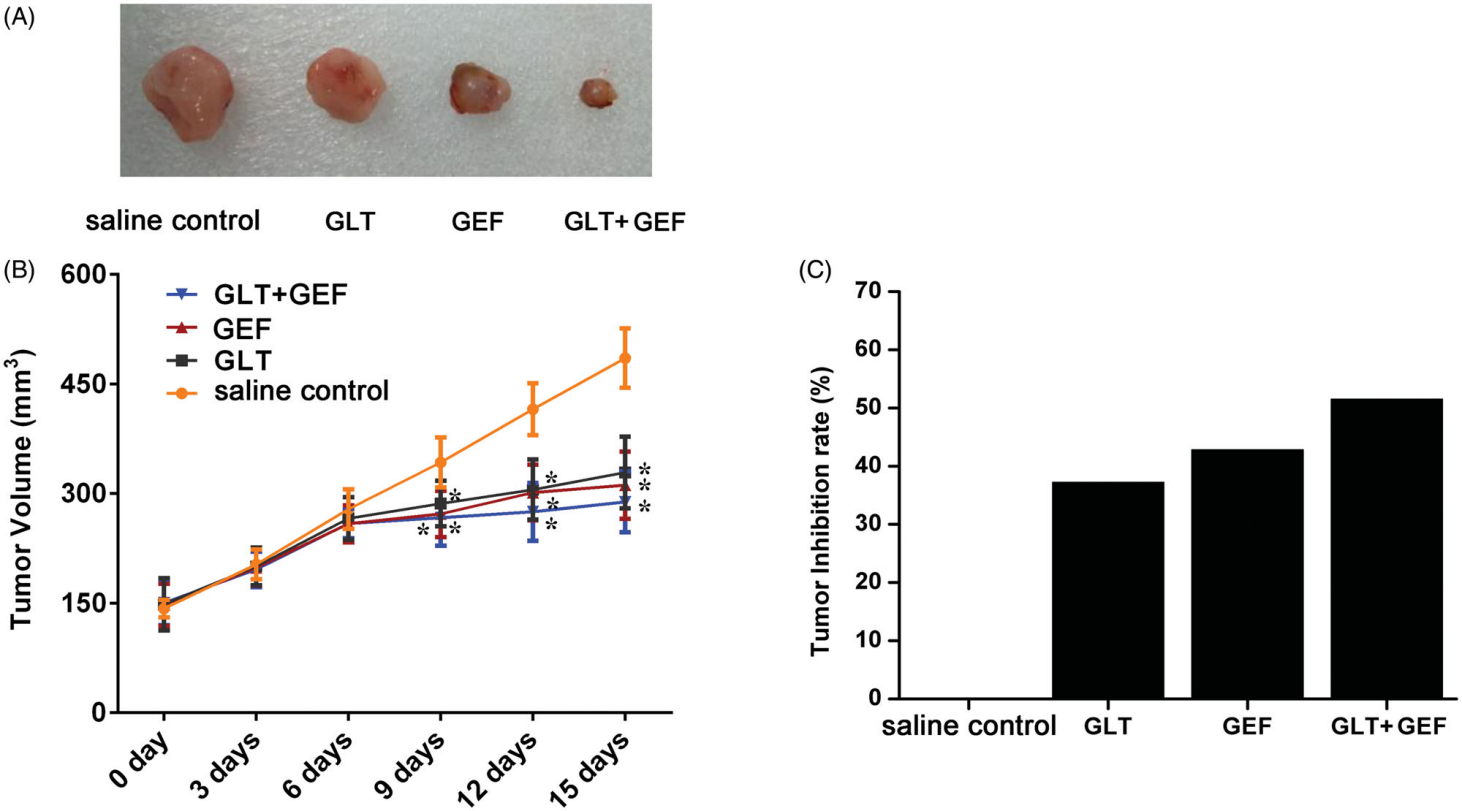
The tumour volume and inhibition rate in each group. (A) The picture of tumour size. (B) The changes of tumour volume; (C) Tumour inhibition rate; *p < 0.05 vs. the saline control group.

The lung cancer A549 cells were inoculated subcutaneously in nude mice, and subcutaneous tumours were formed after three weeks. The diameters of the subcutaneous tumours were approximately 6-8 mm by the calliper. Based on the growth trend in the tumours of each group, they grew rapidly early after administration and grew slowly in the late stage. The results are shown in Figure 2(A,B). There were no significant differences between the groups 6 days after the administration. The tumour volumes in the GEF, GLT + GEF, and GLT groups were significantly lower than that in the saline control group (*p* < 0.05). The results demonstrated that the tumour volume in the GLT + GEF group reduced and that in the GLT group was also significantly lower than that in the saline control group (*p* < 0.05). Moreover, the inhibition rates of the tumours in the GLT, GEF, and GLT + GEF groups were 37.25%, 42.85%, and 51.54%, respectively (Figure 2(C)). It was suggested that the growth of the tumours was inhibited by GEF and GLT, and the effect of GLT + GEF was higher than those of GEF and GLT alone.

### Characteristics of tumour tissues based on H&E staining

**Figure 3.**
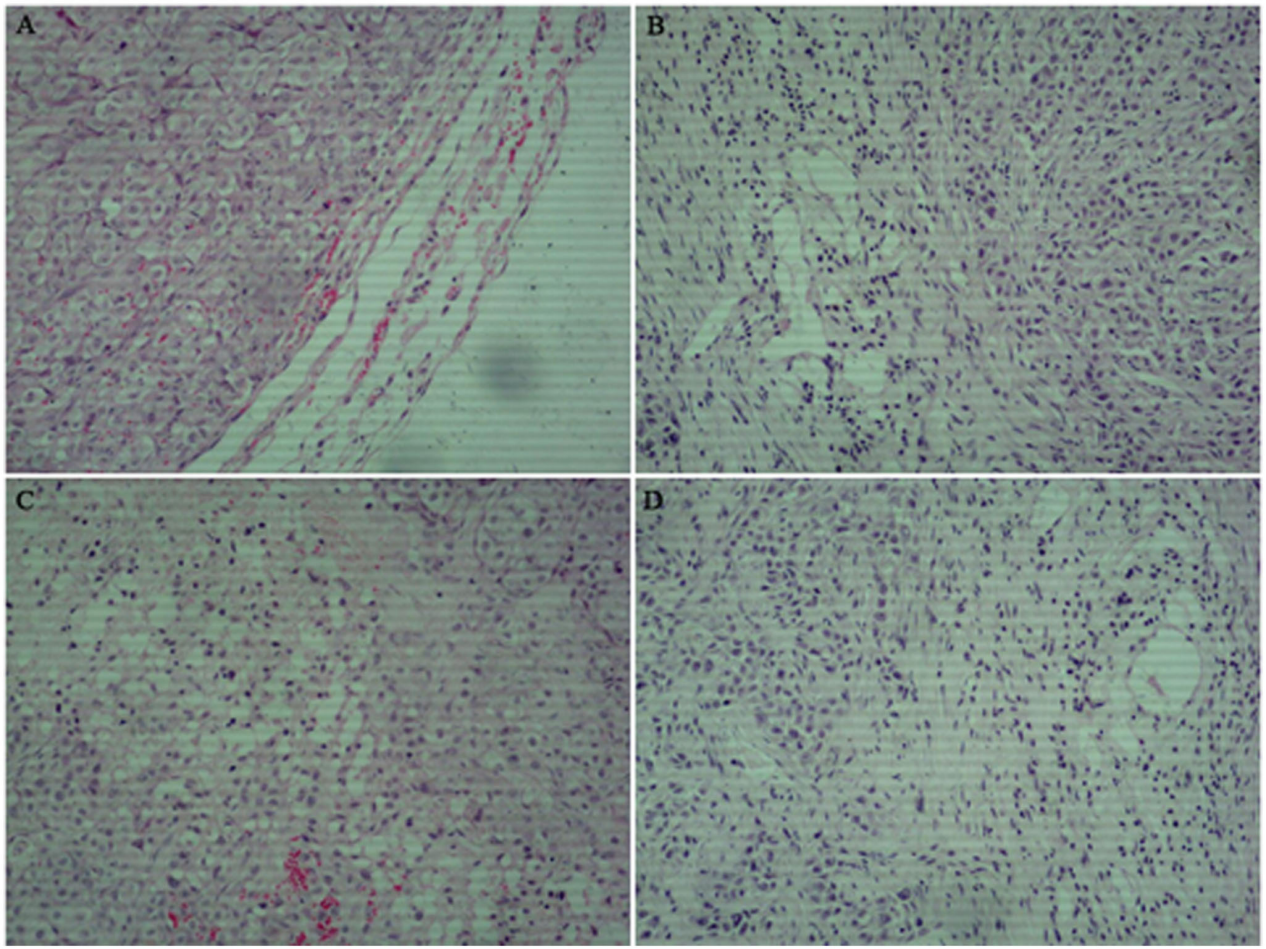
Effects of GLT and GEF on tumour morphology from H&E staining (×100). (A) saline control group; (B) GLT group; (C) GEF group; (D) GLT + GEF group.

H&E staining (Figure 3) revealed that most of the tumour cells in the saline control group showed marked histopathological abnormalities, such as unclear cell boundaries, vacuoles in the cytoplasm, and varying amounts of eosinophilic cytoplasm. Simultaneously, the nuclei of some tumour cells were irregular. The sizes of the nuclei and cells were not uniform. Compared with that in the saline control group, the proliferation of fibroblasts induced by the drugs on the cells was significantly increased in the GEF and GLT groups. Moreover, the infiltration of lymphocytes, plasma cells, and mast cells was decreased in the GLT, GEF, and GLT + GEF groups compared to that in the saline control group. Occasionally, neutrophils and macrophages were distributed throughout the tumour and tumour stroma, and the tumour cells showed interstitial oedema, lymphatic dilatation, and abundant interstitial collagen.

### Changes in tumour tissue MVD

**Figure 4.**
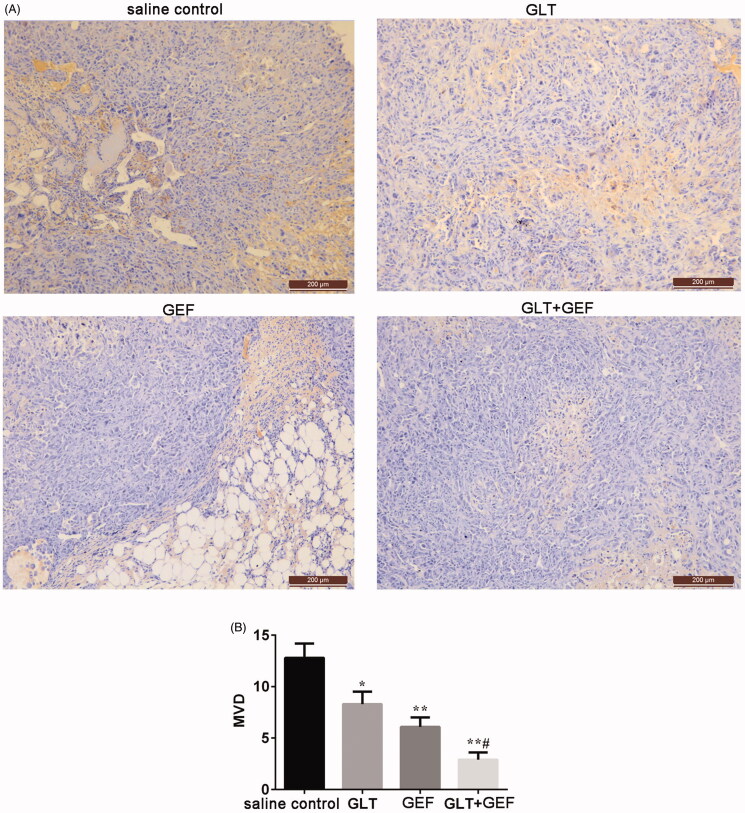
Effect of GLT and GEF on MVD transplanted tumour in nude mice. *p < 0.05 vs. the saline control group; **p < 0.01 vs. the saline control group; *^#^p* < 0.05 vs. GLT group.

CD31-stained tumour tissues were subjected to immunohistochemical experiments to determine the tumour MVD. As shown in Figure 4, the number of blood vessels in the saline control group was 12.8 ± 1.4, and those in the GLT, GEF, and GLT + GEF groups decreased to 8.3 ± 1.2, 6.1 ± 0.9, and 2.9 ± 0.7, respectively (*p* < 0.05). The results indicated that GLTs inhibited tumour angiogenesis, and GLT combined with GEF was more effective.

### Expression of VEGFR2, angiostatin, and endostatin mRNA

**Figure 5.**
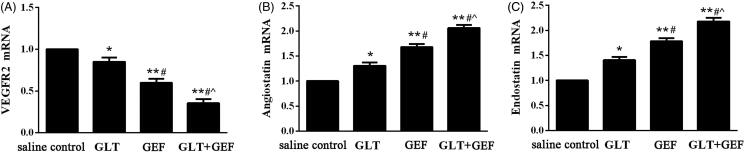
The expression of VEGFR2, angiostatin and endostatin mRNA. (A) the expression of VEGFR2 mRNA; (B) the expression of angiostatin mRNA; (C) the expression of endostatin mRNA.

As shown in Figure 5, compared with that in the saline control group, the expression of VEGFR2 mRNA in the GEF, GLT, and GLT + GEF groups were significantly decreased (Figure 4(A), *p* < 0.05), whereas the expression of angiostatin and endostatin mRNA were significantly increased in the GEF, GLT, and GLT + GEF groups (Figure 4(B,C), *p* < 0.05).

### Expression of VEGFR2, angiostatin, endostatin, bcl-2, and bax protein

**Figure 6.**
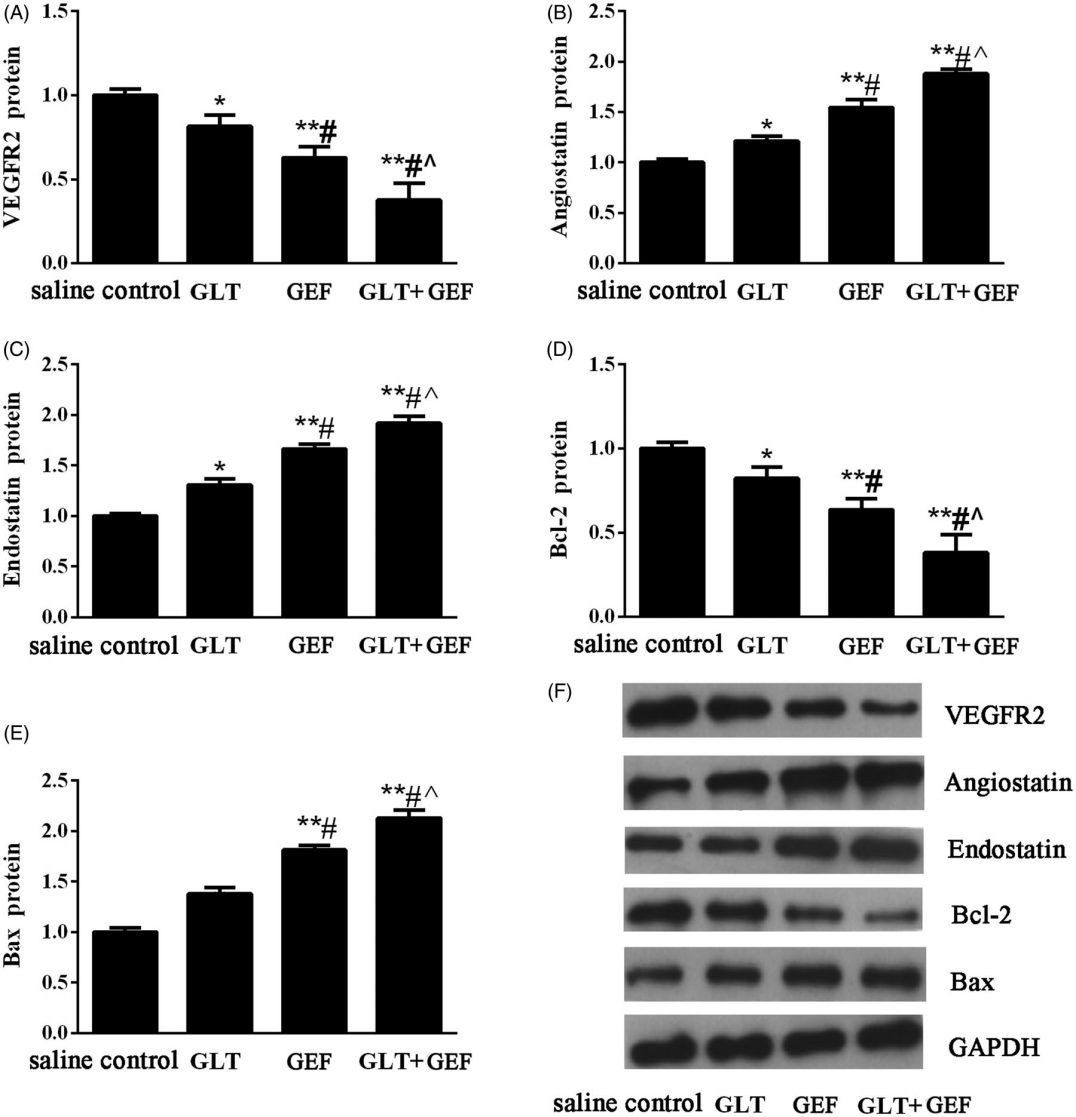
The expression of VEGFR2, angiostatin, endostatin, Bcl-2 and Bax protein. (A) Relative protein expression levels of VEGFR2; (B) Relative protein expression levels of angiostatin; (C) Relative protein expression levels of endostatin; (D) Relative protein expression levels of Bcl-2; (E) Relative protein expression levels of Bax; (E) Representative western blotting images. *p < 0.05 vs. the saline control group; **p < 0.01 vs. the saline control group; *#p* < 0.05 vs. GLT group; ^∧^*p* < 0.05 vs. GEF group.

Compared with those in the saline control group, the expression of VEGFR2 and Bcl-2 protein in the GEF, GLT, and GLT + GEF groups were significantly decreased (Figure 6(A,D), *p* < 0.05), whereas the expression of angiostatin, endostatin, and Bax protein were significantly increased in the GEF, GLT, and GLT + GEF groups (Figure 6(B,C,E), *p* < 0.05). The changes in protein expression in the GLT + GEF group were the most significant. The results indicated that GLT combined with GEF effectively inhibited tumour angiogenesis and promoted tumour cell apoptosis.

## Discussion

In the present study, we found that GLTs inhibited tumour growth in nude mice and promoted tumour cell apoptosis. *G. lucidum* is a widely known mushroom used as traditional Chinese herbal medicine, the characteristics of which have been demonstrated for the treatment of arthritis, cancer, hepatitis, bronchitis, asthma, neurasthenia, deficiency-associated fatigue, and insomnia (Lin et al. 2016). The main extracts of *G. lucidum* including GLTs and polysaccharides have many medical effects and numerous pharmacological uses, such as adjuvant treatment of cancers, hypertension, neurasthenia, hepatitis, and hyperlipidaemia (Zhang et al. 2014). Previous studies have shown that GLTs are associated with many bioactivities such as antioxidative, hepatoprotective, and anticancer activities (Wang et al. 2007). Nguyen et al. (2015) reported the isolation and structural elucidation of GLTs from *G. lucidum* and evaluation of their anti-angiogenic and anticancer effects, which revealed that GLTs display potent anti-angiogenic activity, which may be effective against some human cancer cell lines. VEGFR2 is a receptor-type tyrosine kinase and major signal transmitter of tumour angiogenesis (Gridelli et al. 2017). Angiostatin and endostatin are angiogenesis-inhibiting factors. By inhibiting the proliferation and migration of tumour vascular endothelial cells, they can prevent angiogenesis, reduce primary tumour lesions in experimental animals, and inhibit tumour metastasis (Kubo et al. 2015). In our study, we also found that GLTs showed anticancer effects by inhibiting tumour angiogenesis. The tumour MVD decreased when treated with GLT and GEF, and GLT combined with GEF showed the most effectiveness. The expression of VEGFR2 mRNA and protein decreased after GLT combined with GEF treatments, while the expression of angiostatin and endostatin were increased in saline group. The results suggest that GLTs inhibit tumour angiogenesis. Simultaneously, GLT combined with GEF led to fewer side effects. Tang et al. (2006) reported that *G. lucidum* can cause damage to the mitochondrial membrane of the lung cancer cell line 95-D, thereby triggering cell apoptosis. In this study, we detected Bcl-2 and Bax protein expression; the results showed that GLTs promote tumour cell apoptosis. GLT combined with GEF significantly attenuates tumour cell proliferation.

Lung cancer is the most frequently diagnosed cancers among all the malignant tumours mentioned above. Our results indicated that there are many side effects such as signs of agitation and drowsiness observed in tumour-bearing nude mice treated with GEF, while mice treated with GLTs are no obvious agitation or drowsiness, access water and food without difficulty, and exhibited an ameliorated skin colour. Recent evidence suggests that many biologically active metabolites are effective in terms of their anticancer effects, but these may cause some side effects. Previously, Min et al. (2015) and Hotta et al. (2007) found that a cell cycle-specific antitumor drug, docetaxel, inhibits the depolymerisation of microtubules. Nevertheless, Baker et al. (2008) and de Weger et al. (2014) pointed that docetaxel causes many serious side effects such as nephrotoxicity, cardiotoxicity, and hypersensitivity. Furthermore, berberine, an isoquinoline alkaloid, has been demonstrated to exhibit great anti-tumour effects; however, its hydrophilicity and low absorption in the intestines limit its efficacy for cancer treatment (Xiao et al. 2018). Meanwhile, Amadori et al. (2011) and Seib and Kaplan (2012) indicated that doxorubicin has various antitumor activities accompanied by severe side effects including testicular toxicity and cardiotoxicity. Therefore, in recent years, several natural herbs such as GLTs have been investigated for their sensitization functions and no side effects. Studies have demonstrated that GLTs have anticancer activity, especially in lung cancer (Fatmawati et al. 2011; Cheng et al. 2012). In our study, the CCK-8 assay revealed a dose-dependent loss in viability in GLT- and GEF-treated A549 cells. GLTs showed a much less potent cytotoxic effect in the BEAS-2B cell line, while GEF showed a significant inhibitory action at a high dose. Furthermore, we examined the effects of GLTs in tumour-bearing nude mice. Our results revealed that GLTs inhibited the growth of lung cancer. The results were similar to those of a previous investigation (Chen et al. 2016). Moreover, we found that GLT and GEF reduced the tumour volume and inhibited tumour growth. Meanwhile, H&E staining also revealed that the morphology of tumour cells in the saline control group displayed unclear cell boundaries, vacuoles in the cytoplasm, and varying amounts of eosinophilic cytoplasm. In contrast, mice treated with GLT and GEF resulted in moderate amelioration of inflammatory cell infiltration, lymphatic dilatation, abundant interstitial collagen, and attenuation of the proliferation and migration of tumour cells. GLT combined with GEF showed more effectiveness. Previous studies have shown that GLTs inhibit hepatoma cancer cell proliferation and promote cancer cell apoptosis (Costa et al. 2008; Weng et al. 2010; Lin et al. 2012). Our results were consistent with the results of these studies. In addition, there is no clear evidence to support that GLT combined with GEF inhibits the lung cancer process. Taken together, GLT and GEF combination therapy can significantly inhibit lung cancer proliferation and induce apoptosis. From the perspective of tumour chemotherapy, drug combinations have important clinical significance for reducing the toxicity of antitumor drugs. This result provides a certain reference for guiding drug use in patients with lung cancer.

## Conclusions

The present study demonstrated that GLTs significantly inhibit the growth of lung cancer cells in nude mice. Our study provides evidence that GLT and GEF combination therapy may be a promising candidate for the treatment of lung cancer and as an experimental basis for clinical treatment.
